# Supplementary figures and images for: Development of an instrument to measure mistreatment of women during childbirth through item response theory
Source: PLoS One. 2022 Jul 12;17(7):e0271278. doi: 10.1371/journal.pone.0271278 (PMC9275678; doi:10.1371/journal.pone.0271278)

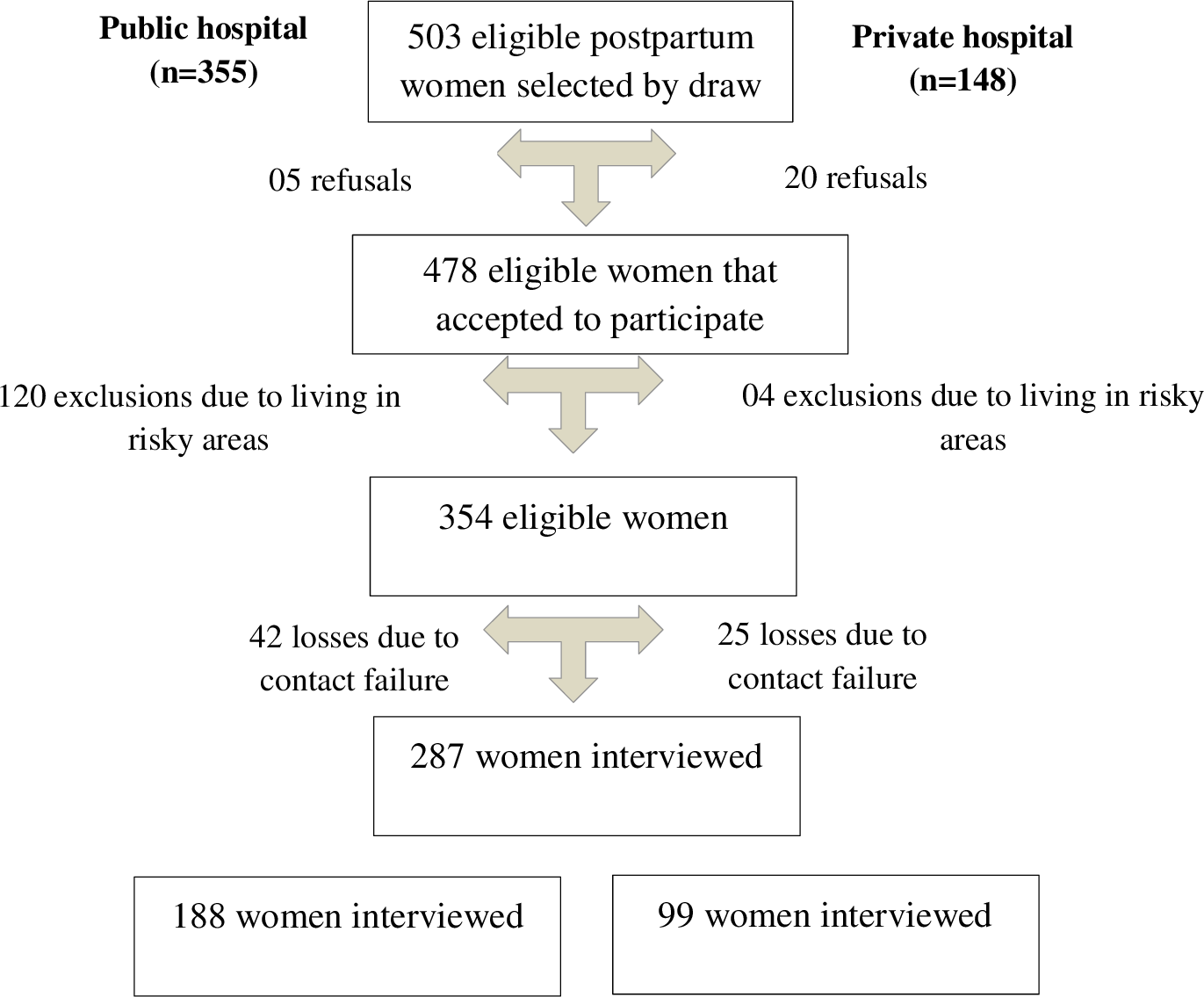

Supplement: S1 Fig — Porto Alegre, 2016. (TIF) [file pone.0271278.s001.tif]
