# Supplementary material for: Development of an instrument to measure mistreatment of women during childbirth through item response theory
Source: PLoS One. 2022 Jul 12;17(7):e0271278. doi: 10.1371/journal.pone.0271278 (PMC9275678; doi:10.1371/journal.pone.0271278)
Supplement: S1 Table — (DOCX) [file pone.0271278.s003.docx]

| **Item** | **Variables tested in models** | **Reason for exclusion** | **Model in which it was included** |
| --- | --- | --- | --- |
| 1 | Had companion during labor | - | Model 1 and 2 |
| 2 | Had companion at delivery | - | Model 1 and 2 |
| 3 | Had companion in postpartum | - | Model 1 and 2 |
| 4 | Felt free to ask questions, clarify doubts, or participate in the decisions | - | Model 1 and 2 |
| 5 | Understood the information provided | - | Model 1 and 2 |
| 6 | Had skin-to-skin contact with the newborn in the delivery room | - | Model 1 and 2 |
| 7 | Felt welcomed in the delivery environment | - | Model 1 and 2 |
| 8 | Felt safe in the delivery environment | - | Model 1 and 2 |
| 9 | Had privacy | - | Model 1 and 2 |
| 10 | Requested analgesia/anesthesia and was not attended | - | Model 2 |
| 11 | Pressure maneuver was performed on the uterine fundus | - | Model 2 |
| 12 | Needed to go to more than one maternity hospital (pilgrimage) | Conceptual issues (explains more mortality than obstetric violence) and for not contributing psychometric information in estimating the latent trait |  |
| 13 | Encouraged to have a companion | Overlaps with variables on caregiver during labor, delivery and postpartum |  |
| 14 | Used pain relief methods | Did not contribute psychometric information in estimation of the latent trait |  |
| 15 | Used non-pharmacological method for pain relief: bath or shower | High number of missing data (valid n:168) |  |
| 16 | Used non-pharmacological method for pain relief: massage | High number of missing data (valid n:168) |  |
| 17 | Used non-pharmacological method for pain relief: Pilates ball | High number of missing data (valid n:168) |  |
| 18 | Used non-pharmacological method for pain relief: hot or cold compresses | High number of missing data (valid n:168) |  |
| 19 | Used non-pharmacological method for pain relief: backrest | High number of missing data (valid n:168) |  |
| 20 | Used pharmacological method for pain relief: orally | High number of missing data (valid n:168) |  |
| 21 | Used pharmacological method for pain relief: by injection | High number of missing data (valid n:168) |  |
| 22 | Used pharmacological method for pain relief: spinal/peridural anesthesia | High number of missing data (valid n:168) |  |
| 23 | Used pharmacological method for pain relief: local anesthesia (perineum) | High number of missing data (valid n:168) |  |
| 24 | Was offered liquids and light food | Did not contribute psychometric information in the estimation of the latent trait |  |
| 25 | Encouraged to walk/wander around | Did not contribute psychometric information in the estimation of the latent trait |  |
| 26 | Performed trichotomy | Did not contribute psychometric information in the estimation of the latent trait |  |
| 27 | Had an enema | Did not contribute psychometric information in the estimation of the latent trait |  |
| 28 | Had oxytocin induced labor | Did not contribute psychometric information in the estimation of the latent trait |  |
| 29 | Amniotomy was performed | Did not contribute psychometric information in the estimation of the latent trait |  |
| 30 | Episiotomy was performed | Did not contribute psychometric information in the estimation of the latent trait |  |
| 31 | Forceps were used (with consent?) | Did not contribute psychometric information in the estimation of the latent trait |  |
| 32 | Chose the delivery position | High number of missing data |  |
| 33 | Newborn stayed in the delivery room with the mother | Conceptual issues, as it overlaps with the item had skin-to-skin contact |  |
| 34 | Explained why the newborn did not stay in the delivery room | Complementary to item 33, which was removed from the analysis for conceptual reasons |  |
| 35 | Felt assisted to start breastfeeding in the delivery room | Did not contribute psychometric information in the estimation of the latent trait |  |
| 36 | The baby was put to the breast within the first hour of life | Excluded because it overlaps with item 35 |  |
