# Supplementary material for: Development of an instrument to measure mistreatment of women during childbirth through item response theory
Source: PLoS One. 2022 Jul 12;17(7):e0271278. doi: 10.1371/journal.pone.0271278 (PMC9275678; doi:10.1371/journal.pone.0271278)
Supplement: S2 Table — (DOCX) [file pone.0271278.s004.docx]

| **Item** | **Response categories** | **MLWC1** | **MLWC2** |
| --- | --- | --- | --- |
| 1. Had a companion during labor | 0. YES  1. NO* | X | X |
| 2. Had a companion at delivery | 0. YES  1. NO* | X | X |
| 3. Had a companion in the immediate postpartum period | 0. YES  1. NO* | X | X |
| 4. Felt free to ask questions, clarify doubts, or participate in the decisions | 0. YES, totally  1. NO** | X | X |
| 5. Understood the information given | 0. YES, totally  1. NO** | X | X |
| 6. Had skin-to-skin contact with the newborn | 0. YES, immediately after delivery  1. NO or after procedures with the newborn | X | X |
| 7. Felt welcomed in the delivery environment | 0. YES, totally  1. NO** | X | X |
| 8. Felt safe in the delivery environment | 0. YES, totally  1. NO** | X | X |
| 9. Had privacy | 0. YES, totally  1. NO** | X | X |
| 10. Pressure maneuver was performed on the uterine fundus | 0. YES (with or without consent)  1. NO |  | X |
| 11. Requested analgesia/anesthesia and was not attended | 0. YES  1. NO |  | X |

* Because it was not allowed or because of a personal situation; ** Little or more or less
