# Supplementary material for: Development of an instrument to measure mistreatment of women during childbirth through item response theory
Source: PLoS One. 2022 Jul 12;17(7):e0271278. doi: 10.1371/journal.pone.0271278 (PMC9275678; doi:10.1371/journal.pone.0271278)
Supplement: Attachment — Submitted filename: Response to Reviewers.pdf [file pone.0271278.s005.pdf]

Porto Alegre, 25 January 2022

Dear Dr. Andreucci,

We were happy to receive your feedback about our manuscript entitled *Development of an instrument to measure obstetric violence through Item Response Theory* [PONE-D-21-31080] and we are thankful for the careful reviews that have been carried out. We have thoroughly and carefully reviewed the manuscript and handled all of the points, one by one. We are sure to be now resubmitting to your appreciation an improved version of our article.

Please find below the point-by-point descriptions and responses to the reviews provided. Each point is addressed with a response in this letter and a description of the amendments that have been made to the manuscript and where in the text they can be viewed. In the manuscript, all amendments are marked in yellow for prompt identification.

Ethics statement has been amended, as well as data availability.

As suggested by all three reviewers, language has been checked by a professional (see attached Declaration) and many changes were made to the text.

- Comments by Reviewer #1:

1. Please describe the settings in which studied women gave birth. I suppose the two facilities are different in many aspects, for example, number of live births per year, health professionals usually responsible for (or involved in) childbirth care, training or teaching activities, c-section rates, etc. Additionally, those who do not know the Brazilian health system and the Brazilian typical obstetric care may find it difficult to understand the public/private contrast.

   Response: We have included the descriptions of the hospitals in the 1st paragraph under Study Design and Population (lines 124-132).

2. I understand the motivation to exclude women "who had formal contraindication for breastfeeding" and those who lived in places considered risky by the research team. However, I wonder if those women are not the most vulnerable ones, and therefore, the most likely to be subjected to obstetric violence. To my view, this is a potential source of bias that should be addressed in the study.

   Response: Concerning the women who had formal contraindication for breastfeeding, we have made it clearer in the text that this was an elegibility criterion (not an exclusion one), because in the original research brestfeeding prevalence was a main outcome. This clarification was made in the 2nd paragraph under Study Design and Population (Lines 134-138). Concerning the women who lived in risky areas, that were actually excluded, we have included a new passage and added references to discuss this as a potential source of bias (before last paragraph of the Discussion – lines: 462-472).

3. "Each day, two women from the public maternity hospital and one from the private hospital were included in the study until the intended sample was reached" (lines 124-126): please, what was the intended sample? How was it calculated? I am afraid I missed this information, and it is essential to discuss the study's limitations.

   Response: We have included a new paragraph (1st paragraph under Sample and Data Collection: lines 143-146) with information about sample size and calculation, with the

citation of the former research that originated the present study. We have also included in the last paragraph of the Discussion more information about the sample size needed to adjust an IRT model, with a new reference. This way, the study's limitations concerning the sample size are clarified.

4. All information was collected during face to face interviews? How was socioeconomic level assessed? Please explain the categories' grouping.

   Response: All information was collected during face to face interviews. This has been made clearer in the text, in the third paragraph under Sample and Data Collection (lines 160-165). In this same paragraph, we have explained how socioeconomic level was assessed and it's categories grouped.

5. Maybe you could attach a supplementary figure, or table, in order to clarify the number of women drawn in each hospital, the ones who were eligible, included, interviewed, etc. This would be an opportunity to clarify non-participation at each stage, for instance, the refusals and women who were excluded because they lived in a territory assessed as risky by the research team (it is not clear when or how this evaluation and exclusion was made).

   Response: A flowchart was included as supplementary Figure 1, cited in the manuscript in the 1st paragraph under Results (line 243).

6. Please, clarify why there are missing data (table 1).

   Response: The reasons for missing data were clarified with footnotes in Table 1.

7. I would strongly suggest that you add a table with more detailed information about women's outcomes, such as c-section rates, according to key characteristics, such as by type of hospital (public x private), women's skin color, etc. From previous studies we can suppose these results will have an impact in women's perception of mistreatment. Additionally, it is not clear if women who went into labor but had a c-section were included in the "women who went into labor" group or not.

   Response: The women who went into labor but had a c-section were included in the "women who went into labor" group. This information was made clearer in the second paragraph under Results. Also, Table 2 was added, presenting c-section rates and proportion of women who went into labor according to key characteristics. The findings of this new table are described in the Results session and some of them are discussed in a whole new paragraph in the Discussion (6th paragraph under that session), with new reference citations.

8. In the same direction, I would suggest that you add some categories in table 1, for instance, women who did not use pain relief methods. Did they feel they were subjected to obstetric violence? I am eager to know that! Moreover, it was a bit difficult to me to understand why some categories showed were recommended practices ("encouraged to walk", "yes"), while others were the practices that should be abolished ("enema", "yes").

Response: We have included in Table 1 the complementary category of each variable related to the care of women who went into labor. We believe this clarifies the issue, making it easier to understand those interventions and situations in light of being recommended or not.

9. In the discussion section, I would suggest to include some thoughts on the data collection strategy. The face to face interview made outside the health facility may have advantages and disadvantages.

   Response: We have included some thoughts on potential disadvantages of face to face interviews in the before last paragraph of the Discussion (lines: 459-462).

10. I would suggest you explore more deeply the feelings of unsafety reported by women. The hospitalization of childbirth care is often justified as the historical "evolution" of the obstetric assistance, understanding it as a pathway to safer care for women and babies. However, it seems that women do not agree with this. Additionally, we suppose that the health facility exists to provide care, but what does it mean when those who should be cared for say they don't feel welcomed? These reflections could reinforce the need for further studies on obstetric violence and the possibilities to measure it.

    Response: A new paragraph was added in the Discussion, with new reference citations, exploring this issue. This is the 10th paragraph under Discussion (lines: 441-452).

11. Maybe you could use the STROBE checklist before submitting the revised manuscript.

    Response: The STROBE checklist was used and added as a supplementary file.

- Comments by Reviewer #2:

1. According to Strobe Checklist, Introduction and Abstract are adequate. Methods sections would be improved if authors include a brief statement of any efforts carried on to address potential sources of bias; and also if they describe how data was collected, registered and organized. Results and discussion are well described.

   Response: We have included a new paragraph describing how potential sources of bias were addressed, as well as included more details about data collection, registering and organization. This is the 5th paragraph under Sample and Data Collection (lines 171-178).

2. I suggest some background information update as the manuscript refers to "Rede Cegonha" which is now not available anymore, as it is not a public policy but a governamental program/project.

   Response: We have reviewed updated information about "Rede Cegonha" and other programs related to prenatal and childbirth care in Brazil. We have modified the 4th paragraph of the Introduction (Lines 75-94) and inserted new sentences with updated references. In this review process we were able to certify that "Rede Cegonha" is still available and adherent institutions send their indicators monthly for monitoring. The ordinance supporting the program today is: Consolidation Ordinance n.3, 28 September 2017 (available at:

https://bvsms.saude.gov.br/bvs/saudelegis/gm/2017/prc0003_03_10_2017.html#ANEXOIITITIICAPII). Even though the program still exists, it has clearly weakened in terms of institutional strength, so we have included this information to clarify the context.

3. Also, the findings that led to Itens exclusion as described on Supplementary Table 1 are very interesting and maybe authors could consider including one paragraph about those itens on the manuscript results and discussion, as they bring along interventions and situations that are highly prevalent in the country.

   Response: We have drawn more attention to episiotomy and amniotomy, which are very frequent practices in our country. We have included new passages in the 3rd paragraph under Results and in the 5th paragraph under Discussion. Also, the paragraph beginning with “The gap between the perception of violence” mentions some of the findings of Supplementary Table 1.

- Comments by Reviewer #3:

1. "Humanized" and "obstetric violence" are not the terms used by international researchers. I understand that these are terms used mainly in Brazil and Latin America, but considering that this article will be read by international readers, and that there are already well-known terms for both, it is important to use them or explain to the reader why you chose to use this definition instead of the well-established one.

   Response: we have reviewed these terms throughout the whole text. The term “obstetric violence” was replaced with “mistreatment of women during childbirth”. The term “level of obstetric violence” was replaced with “mistreatment level of women during childbirth”. Therefore the acronym NVO changed into MLWC.

2. Although I am not an expert in quantitative studies, it seems to me that the statistical aspects are well described. On the other hand, I missed more details about the questionnaire in the Data Collection section.

   Response: We have provided more details about the questionnaire in the 3rd paragraph under Sample and Data Collection (lines 158-160).

3. Lines 322 to 336 are presenting Results instead of Discussion. Consider moving up.

   Response: These lines were moved up to the Results session (lines: 256-259) and some remaining passages in the Discussion were adjusted accordingly.

We believe we have fully addressed all the points raised by the reviewers and we thank you again for the opportunity of qualifying our manuscript.

Yours sincerely, the author.
