# Supplementary material for: Development of an instrument to measure mistreatment of women during childbirth through item response theory
Source: PLoS One. 2022 Jul 12;17(7):e0271278. doi: 10.1371/journal.pone.0271278 (PMC9275678; doi:10.1371/journal.pone.0271278)
Supplement: Attachment — Submitted filename: Response to Reviewers.docx.pdf [file pone.0271278.s006.pdf]

Porto Alegre, 09 June 2022

Dear Dr. Andreucci,

We were happy to receive your second feedback about our manuscript entitled *Development of an instrument to measure obstetric violence through Item Response Theory* [PONE-D-21-31080] and we are thankful for the careful reviews that have been carried out. We have once more handled all of the points, and we are thus resubmitting to your appreciation an improved version of our article.

Please find below the point-by-point descriptions and responses to the reviews provided. Each point is addressed with a response in this letter and a description of the amendments that have been made to the manuscript and where in the text they can be viewed. In the manuscript, all amendments are marked in yellow for prompt identification.

- Comments by Reviewer #1:

1. I would like to bring up a few more questions just to deepen the discussion around obstetric violence scholarship. Indeed, I believe "obstetric violence" has a concept of its own, and it has gained ground in academic studies from various disciplines in the last decade, as can be verified from the growing number of articles published using this term. In my view, the use of "obstetric violence" poses a political stance, as it implies the recognition of structural problems that negatively affect women's experiences, jeopardizing their human rights.

I understand you changed to "mistreatment" to meet one of the reviewer's request, however, I was wondering which term or concept you used in the Brazilian Portuguese questionnaire, as I feel there may be differences in understanding between them. Unfortunately, I couldn't access the file "database_02_22.xlsx" to verify this. In any case, I would suggest that you reflect about the use of terms in your empirical material and in the manuscript before making a final decision between "obstetric violence" and "mistreatment".

Response: We are totally aligned with the view of this Reviewer. Although we have decided to keep the term "mistreatment" in the majority of the text, we have included the term "obstetric violence" in a few points, such as in the abstract, in order to point to the wider context reflected by this term, its poltical stance and the recognition of the structural issues involved. We have also included the term "obstetric violence" where the original study cited used this same term on its data collection. Our decision to keep the term "mistreatment" was for the following reasons: In the Brazilian Portuguese questionnaire we used the following question: "Have you ever (during labor and childbirth care) felt disrespected, humiliated or mistreated by health professionals?", we have not actually used the term "obstetric violence"; the term mistreatment is more frequent in the international literature, so the use of this term can increase access and visibility of the article. Considering the relevance of the term "obstetric violence", we decided to add a paragraph in the Introduction (lines 71-77).

2. You mention that "All information considered in this study was collected during face-to-face interviews and the responses were referred by the participants. Women who were not found for the interview, after at least three attempts of contact by telephone and one in person, were considered a loss". However, in the results, you state that "The women not interviewed due to losses and refusals differed in terms of education and skin color,

showing less education ($p<0.01$) and a higher prevalence of white skin color compared to those interviewed ($p=0.032$)". How did you get information about education and skin color from women who refused to participate?

Response: Name, age, education and skin color were drawn from the hospital chart, so these were the only data available for losses and refusals. We modified the text for clarification (Lines 169-171).

3. Table 1, you added the information that "*Missing data correspond to responses 'I don't know' or 'I don't remember'" – for some variables, would it be relevant to consider these answers? For example, for "Induction with oxytocin", when women don't know whether or not they underwent this procedure, is it possible to state that they didn't receive appropriate information? Can we say the same about "would like another position in labor"? If 54 women answered "I don't know" or "I don't remember", it means that more than 25% of the birthing women didn't receive adequate information about mobility during labor – however, this variable was excluded from the model because of the high number of missing data. I don't mean that you have to re-process your data, but then again, I wonder what does it mean when we face such a high level of women who declare that they do not know if they would like another position in labor. It seems to me that they lack information about their rights, options and about evidence, as well.

Response: We agree with the reviewer's suggestion and have therefore included a paragraph in the manuscript to briefly discuss these findings (lines 444-448).

- Comments by Reviewer #2:

1. I believe an editorial and authorial decision will be need in order to comprehend or not the very recently published "PORTARIA GM/MS Nº 715, DE 4 DE ABRIL DE 2022" by Brazilian Ministry of Health, which alters the Rede Cegonha program into Rede de Atenção Materna e Infantil (Rami).

   Response: We have included the recent "portaria 715/2022", which alters the Rede Cegonha program, briefly presenting this still very recent context change (lines: 92-101).

We believe we have fully addressed all the points raised by the reviewers in this new round and we thank you again for the opportunity of qualifying our manuscript.

Yours sincerely, the authors.
